# Assessment of Water Footprints of Consumption and Production in Transboundary River Basins at Country-Basin Mesh-Based Spatial Resolution

**DOI:** 10.3390/ijerph16050703

**Published:** 2019-02-27

**Authors:** Xia Wu, Dagmawi Mulugeta Degefu, Liang Yuan, Zaiyi Liao, Weijun He, Min An, Zhaofang Zhang

**Affiliations:** 1 School of Law and Public Administration, China Three Gorges University, Yichang 443002, China; ctguwuxia@163.com; 2 College of Economics and Management, China Three Gorges University, Yichang 443002, China; weijunhe@ctgu.edu.cn (W.H.); anmin@hhu.edu.cn (M.A.); 3 Faculty of Engineering and Architectural Science, Ryerson University, Toronto, ON M5B 2K3, Canada; 4 School of business, Hohai University, Nanjing 210098, China; zackzhang@hhu.edu.cn

**Keywords:** virtual water, water footprint, transboundary river basins, blue water, green water, gray water

## Abstract

Water is unevenly distributed globally. This uneven distribution is the reason behind the differences among geographical areas in terms of their water footprint of consumption and production. This gives the global trade of goods a unique feature. This characteristic of the water footprint might be used to address water scarcity and conflicts because water availability also has the same trend. Transboundary river basins are freshwater resources with a high probability of water scarcity and conflict because the water is claimed by multiple sovereign countries. In order to design sharing mechanisms for transboundary river basins that incorporate virtual water concept, it is key to identify the virtual water balance of country-basin units. A study addressing this research gap is not yet available. This article identified and discussed net virtual water importer and exporter sub-basins of transboundary rivers at a country-basin mesh based spatial resolution. The results of our study show that out of the 565 country-basin units surveyed in this article 391, 369, and 461 are net gray, green, and blue virtual water importers respectively. These sub-basins covers 58.37%, 47.52% and 57.52% of the total area covered by transboundary river basins and includes 0.65, 1.9, and around 2 billion people, respectively. The results depict that not only the water endowment of sub-basins is a determining factor for their water footprint of consumption and production, but also their social, economic, and demographic profiles. Furthermore, the water footprint of consumption and production within most of the country-basin units have a global feature. Hence, sustainable water management schemes within border-crossing basins should take into account not only the local but also the global water footprints of consumption and production. This can offer more options for sharing transboundary river basins water capital, thereby minimizing the probability of water scarcity and water conflicts.

## 1. Introduction

Water is one of the most important renewable natural capital that sustains social-economic-environmental systems. This essential resource is unevenly distributed in different forms. Most of the global water exists as salt water (97.5%) and only the remaining 2.5% is available as freshwater. Out of the total freshwater, only 31.1% is available in aquifers, lakes, and rivers for human consumption. The rest is locked in the ice caps [1]. Furthermore, the water available for human consumption is spatially and temporally unevenly distributed [2].

One of the ways this global spatial and temporal uneven distribution of water is mitigated is through virtual water trade. Virtual water is the water used to produce goods and services [3,4]. Virtual water or exogenous water increase the water available to an area. Because water can be allocated from water abundant areas to water-scarce areas through water-intensive goods and services. Hence, in a way, it plays a role in mitigating water shortage throughout the world. For this reason, calculating virtual water balance is very important to get insights into the water footprint of any geographical area [5]. Furthermore, understanding the temporal and spatial variation of virtual water footprints of consumption and production for a specific area is important to design a sustainable water management scheme that takes in to account not only the local water footprint and availability, but also the global water footprint and availability. This can help to solve water scarcity problems [6].

Taking this into consideration in order to give a global picture of virtual water balance and identify the regions which are virtual water exporting and importing various researchers performed assessments at different spatial and temporal resolutions. Hoekstra and Hung (2002) [7] did the pioneering work on global virtual water balance assessment at the national level. After their work Hoekstra and Chapagain (2007) [8]; Chapagain and Hoekstra (2008) [9]; Chapagain et al. (2006) [10]; Mekonnen and Hoekstra (2010) [11]; Hoekstra and Chapagain (2008) [12]; Fader et al. (2011) [13] and Zhan-Ming and Chen (2013) [14] did more comprehensive assessments of water footprints of production and consumption globally.

As much as conducting an analysis of virtual water balance globally at the country level is important understanding virtual water balance of river basins is also vital for river basin management. Acknowledging this need Dumont et al. (2013) [15]; Vanham (2013) [16]; Vanham and Bidoglio (2014) [17]; Zeitoun et al. (2010) [18]; Zhuo et al. (2014) [19], and Feng et al. (2012) [20] conducted their studies at the river basin scale. Even though these studies provided insights at basin resolution, they fail to capture the water footprint of consumption and production differences among the rivers’ sub-basins. This research gap needs to be addressed especially when rivers are transboundary. The following are the main reasons why. (1) These river basins’ water rights are bones of contention among their different riparian countries. As the result water disputes could arise within these basins. Virtual water can be one of the tools used to solve water sharing problems in these river basins. (2) The existing 276 river basins are located within 151 countries, include around 42% of the world’s population, cover 42% of the total surface area of the Earth, and produce roughly 54% of the global river discharge [21,22]. These river basins export and import water as virtual water. As a result, the basins are either net virtual water dependent on other river basins or other river basins are net virtual water dependent on them. When we take this into consideration the number of stakeholders to the problem of water sharing in most of the transboundary rivers are not only the riparian countries but also, indirectly, the regions which depend on them to obtain their water-intensive products. Hence, water issues within these river basins affect regions which are located outside the basins, as well. These are the reasons why having a deeper understanding of the blue, green, and gray virtual water consumption and production differences among these basins’ country-basin units is essential.

Previous studies at the river basin scale overlooked the following aspects: (1) They are not done exclusively for transboundary river basins and at country-basin mesh spatial resolution. Studying the water footprint of consumption and production at country-basin sub-basin resolution is vital because water management policies being implemented within country-basin units are different and sub-basin specific. Therefore, we need to have insights into the consumption and production water footprints of these sub-basins for designing fair, efficient and basin-wide water management systems. (2) Past studies considered the total virtual water consumption and production rather than disaggregating the total water footprint into blue, green, and gray net virtual water balances. Determining the blue, green, and gray virtual water balances is important information if we are to plan sustainable water management policies which are consistent with the basins socio-economic and environmental settings [16,23]. (3) Most of the virtual water balances assessments in the past are done at the river basin or national spatial scales. Hence global study at country-basin mesh spatial resolution is lacking. In this paper acknowledging the role virtual water could play for the sustainable management of transboundary river basins the authors presented a virtual water balance assessment of global transboundary river basins at a spatial resolution of country-basin unit. The main objective of this article is to identify the country-basin units that are blue, green, and gray virtual water importing and exporting. In addition, will also provide a glimpse into the asymmetries among the country-basin units and their significance.

## 2. Materials and Methods

The map of country-basin units was obtained by meshing raster maps of political boundaries and transboundary river basins of the same resolution. Raster maps of country and basin boundaries were obtained by converting country and basin polygon maps from the Transboundary Waters Assessment Program (2016) [21] to 30 arc-minute resolution. UN-adjusted population count, which is an estimate of the number of persons per 30 arc-second (~1 km) grid cell for the year 2000, was obtained from Center for International Earth Science Information Network—CIESIN—Columbia University (2015) [24] and was resampled to 30 arc-minute resolution.

**Figure 1:**
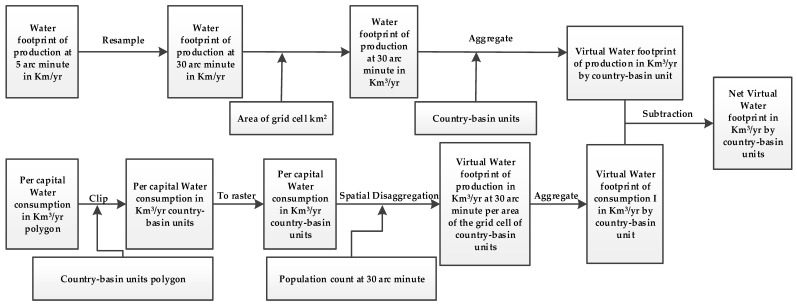
A framework for calculating the virtual balance within global transboundary river basins at a spatial resolution of the country-basin units.

In order to determine the net virtual water footprint of any region or area, its water footprint should be disaggregated into water footprints of consumption and production (see Figure 1). The net virtual water was defined as the difference between the amount of water footprint imported and exported or the difference between the water footprints of consumption and production of a certain geographical area. The water footprint of consumption is the amount of water used to produce the direct and indirect products consumed by a certain geographical region [11]. The water footprint of production is the amount of water used by an area or a region to produce goods for internal or external consumption [11].

The water footprints of production for blue, green, and gray waters were calculated by aggregating the global water footprint obtained from Hoekstra et al. (2012) [25] at 30 arc-minute resolution to a country-basin spatial resolution. These water footprints were calculated by summing agricultural, industrial, and domestic blue, green, and gray water uses. The agricultural water footprints of production were calculated by estimating the water used by crops over their growing period. The domestic and industrial water footprints were obtained by distributing the national water footprint data from the World Bank according to the population density from Center for International Earth Science Information Network—CIESIN—Columbia University (2015) [26].

Ten year average of annual blue, green, and gray per capita water consumption data were obtained from Mekonnen and Hoekstra (2010) [27]. Water consumption per country-basin unit was determined by disaggregating the national per capita water consumption data using the population count of the sub-basins. Then it was aggregated to the country-basin unit.

## 3. Results and Discussion

**Figure 2:**
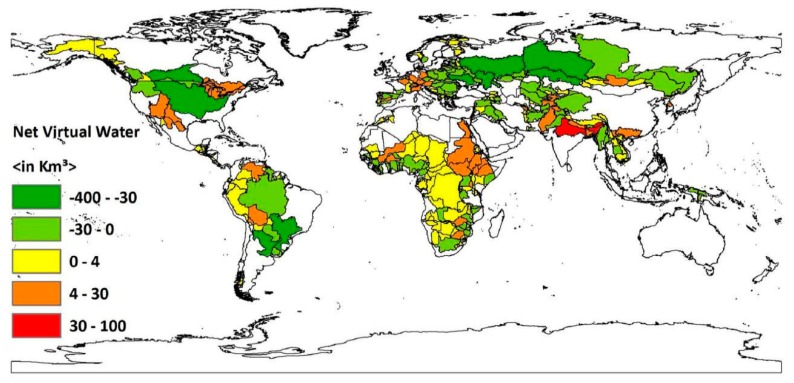
Net virtual water balance for global transboundary river basins’ country-basin units. Period: 1996–2005. This map was generated with ArcGIS 10.2 for desktop from Environmental Systems Research Institute (ESRI), Redlands, CA, USA [28].

A country-basin unit is either a blue, green, and gray water importing or exporting sub-basin. This is determined by its socio-economic and environmental features. Out of the total country-basin units of transboundary river basins surveyed in this article 361 have water footprints of consumption greater than their virtual water footprint of production during the study period. Therefore, the majority of the global country-basin units are net virtual water importers. These sub-basins cover around 2804.5 million km^2^ and include 1767 million people, which is around 70.4% of the total population within the transboundary river basins assessed in this paper. From the net virtual water import map, it is difficult to identify either transboundary rivers’ country-basin units are blue, green, and gray water importing or exporting sub-basins (see Figure 2). Therefore, the total virtual water balance of these sub-basins should be further disaggregated into blue, green, and gray virtual water footprints of consumption and production (see Figures S1–S7, Tables S1–S3 and Appendix A). Doing this could provide insights which are important for more practical water management [16]. Mainly because the water footprints of consumption and production of the sub-basins are consistent with their socio-economic and environmental profiles.

**Figure 3:**
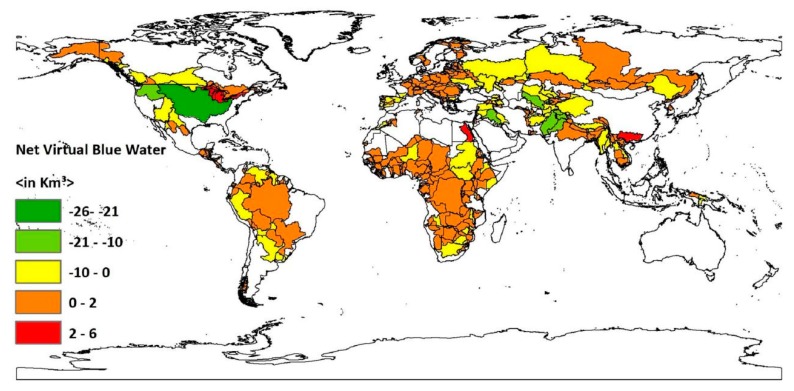
Net blue virtual water balance for global transboundary river basins’ country-basin units. Period: 1996–2005. This map was generated with ArcGIS 10.2 for desktop from Environmental Systems Research Institute (ESRI), Redlands, CA, USA [28].

The blue virtual water balance values for global transboundary river basins’ country-basin units indicated that 461 out of the 565 country-basin units studied in this article are net blue virtual water importers (see Figure 3 and Table 1). These sub-basins include around 2 billion people and cover an area of 3681 million km^2^. Whether a sub-basin is blue water importer or exporter depends not only on the availability of high runoff but also on various socio-economic and demographic features conditions of the sub-basins such as population number or/and density, urbanization level, economic activity, and water consumption behavior. The Senegal River’s sub-basin in Mauritania and Niger River’s sub-basin in Algeria are examples of country-basin units which might be net importers of blue virtual water because they are located in arid regions [29,30]. High population number and/or density, as well as high water consumption behavior, are also the main reasons for a number of transboundary river basins’ country-basin units being net blue virtual water importers. The lower Nile in Egypt and the Limpopo river’s country-basin units in Southern Africa, the Tagus/Tejo country-basin units in Spain have high population count or/and density [31,32,33]. For instance, the Tagus country-basin unit in Spain is the main water source for various sectors: over six million people, most of them in the Madrid area, are dependent on this sub-basin [34]. This might be one of the main reasons for the positive blue virtual water balance of the country-basin unit. Some transboundary river basins’ country-basin units are net blue virtual water importers even though their river discharge or runoff is very high. Among these sub-basins, the main ones arethe St. Lawrence in the United States, the Bei Jiang/Hsi in China, the Amazon in Brazil, Colombia, Venezuela, and Bolivia, the Niger in Nigeria, and the Mekong in Thailand and Cambodia. Lack of economic capacity to develop the resources, high consumption behavior, and high population number might be the reasons for these sub-basins being net blue virtual water importers. For instance, in the Limpopo river basin in Southern Africa in addition to the population increase, lagging water infrastructure development is the reason for the basin being blue virtual water importer [35]. The St. Laurence country-basin unit in the United States of America is a highly urbanized area [36], hence it has a higher blue water consumption footprint than blue water production footprint. Country-basin units with negative blue virtual balance are usually characterized as being rural areas with high agricultural production and lower domestic as well as industrial consumption [16]. The Columbia River Basin in the United States of America is an example of country-basin unit with high river discharge and negative net blue virtual water balance. The river is highly utilized for agricultural purposes [35] Agriculture is the sector that has a large blue water footprint compared to other sectors [11].

**Figure 4:**
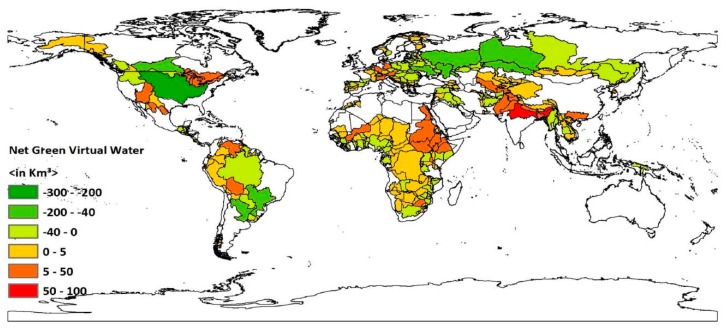
Net green virtual water balance for global transboundary river basins’ country-basin units. Period: 1996–2005. This map was generated with ArcGIS 10.2 for desktop from Environmental Systems Research Institute (ESRI), Redlands, CA, USA [28].

The results for green virtual water balance within transboundary river basins at this spatial resolution showed that the values for 369 country-basin units out of the 565 are below zero (see Figure 4 and Table 1). Hence the majority of them are net green virtual water importers. These river sub-basins cover an area of 3041.25 million km^2^ and include around 1882.03 million people.

The green virtual water footprint of consumption and production is mainly determined by the level of precipitation, the extent of urbanization, the population number or/and density and agricultural activity within the country-basin units. Some regions are net importers of green virtual water even though they have the water resources and the economic capacity to increase production and become net exporters of green virtual water. These basins have positive green virtual water balance not by necessity rather by choice since they have ample green water capital to increase production [27]. Basins in Northern Europe are notable examples. The river basins in this region are characterized by highly urbanized areas, where there are no high water consumption agricultural areas. Other country-basin units which are net virtual green water importers even though they receive a high level of precipitation are the Ganges-Brahmaputra-Meghna’s country-basin units, country-basin units of the Amazon in Peru and Columbia, the Orinoco in Venezuela and Cambodia, country-basin units of the Congo, the sub-basin of the Nile in Ethiopia, and the Bei Jiang/His in China. The reasons for these river basins being net green virtual water importers might be the lack of economic capacity to expand production or huge consumption trend as the result of the high population count/density [33,35,37]. The other reason might be because these sub-basins rely more on irrigated agriculture than rain-fed agriculture. Negative net green virtual water values are associated with net green virtual water export. Sub-basins of the Ogooue in Gabon, the Niger in Nigeria, the Nile in Uganda, the Amazon and La Plata in Brazil and Argentina, and sub-basins of the Mekong, except for the river’s sub-basin in Cambodia, are the sub-basins which have a high level of precipitation and negative green virtual water footprint balance. Most of these country-basin units are a food production unit through rain-fed agricultural practices [38,39,40]. For example, the Nile in Uganda has high and reasonably well-distributed rainfall and conducive areas for agricultural development [41].

**Figure 5:**
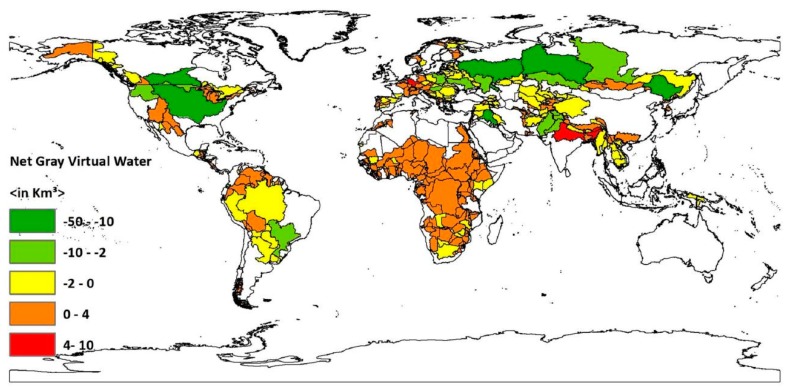
Net gray virtual water balance for global transboundary river basins’ country-basin units. Period: 1996–2005. This map was generated with ArcGIS 10.2 for desktop from Environmental Systems Research Institute (ESRI), Redlands, CA, USA [28].

Transboundary river basin’s country-basin units which have net gray water footprint out of their boundaries are 391 (see Figure 5 and Table 1). Their area coverage is 3735 million km^2^ and has a population count of 1860.08 million people, which is 74.13% of the total population within transboundary river basins. On the other hand, those which are net gray water footprint exporters are 174 in number. These country-basin units cover 3735 million km^2^ and are inhabited by more than half a billion people. Country-basin units of the Ganges-Brahmaputra-Meghna in India and Bangladesh, the Bei Jiang/His and the Mekong in China, the Aral Sea in Kyrgyzstan, Turkmenistan, and Tajikistan, and most of the country-basin units of transboundary rivers in Western Europe are the sub-basins which have net pollution footprints located outside their boundaries. These river basins are characterized by high population density and water-intensive agricultural production. For instance, in the Ganges-Brahmaputra-Meghna in India and Bangladesh, population density is very high [33]. While the Zambezi in Angola and Mozambique, the Orange in Botswana and South Africa, the Okavango in Zimbabwe, the Niger in Guinea, Ivory Coast, and Burkina Faso, the Tigris-Euphrates/Shatt al Arab’s country-basin units, and the Guadiana in Spain, the Garonne in France, and most of the transboundary river basins’ country-basin units of Eastern Europe are net gray virtual water exporting river sub-basins. These country-basin units are characterized by high agricultural activity [29,42,43,44,45,46]. Since the majority of traded virtual water is embedded within agricultural products [27], it is justifiable that these country-basin units are net gray virtual water exporters. Agriculture is not only the major water-consuming sector but also polluting, too [11,25].

In this research article, the authors tried to show the difference among the sub-basins of the global transboundary river basins in terms of their virtual water footprints consumption and production at country-basin mesh spatial resolution. Even though the article identifies blue, green, and gray water importing and exporting country-basin units, it needs to be updated through further research to address the following research gaps. (1) The blue, green, and gray water consumption and production vary through time and space, hence this research should be updated for current and future time periods. (2) The amount of blue, green, and gray water which is exported or imported to and from a country-basin unit should be disaggregated further to quantify the domestic and international virtual water trade. (3) How blue, green, and gray virtual water footprints of consumption and production differences among country-basin units can be used for solving the water sharing problem within transboundary river basins is also an area that is left for future study. Generally, the authors hope the insights from this article can assist to design mechanisms for the sustainable management of transboundary river basins.

## 4. Conclusions

The socio-economic and environmental features of different geographical areas are different. As the result, their blue, green, and gray water consumption and production footprints are also not the same. This difference also exists among the sub-basins of transboundary river basins at the country-basin mesh spatial resolution. Transboundary river basins cross political borders, cover a large area, includes billions of people, and are responsible for more than half the global river discharge. Hence their significance is indisputable. Water sharing problem might arise within these basins at times of water scarcity and could affect the socio-economic and environmental setups within the basins. Furthermore, the problem could be felt outside these basins because these rivers may also have stakeholders that are indirectly water dependent on them through the virtual water. This article identified the net virtual water importers and exporters within global transboundary river basins at a country-basin mesh spatial resolution. The main motivation for this study is the hope that such information might be used for solving water sharing problems by finding ways to link virtual water of consumption and production within and outside of these rivers’ boundaries.

The results of our study showed the differences among the sub-basins of transboundary river basins in terms of virtual water consumption and production. Based on the results the following main conclusions can be drawn. (1) Generally, the blue, green, and gray virtual water consumption and production footprints within most of the transboundary river basins are highly influenced by their social, economic, and demographic characteristics not only by their discharge amount and precipitation level. (2) The blue, green, and gray water footprints of production and consumption vary greatly within most of the transboundary rivers’ country-basin units. (3) Water can be shared by linking the issues based on the blue, green, and gray water consumption and production trends within the basins. (4) The population number within blue, green, and gray virtual water importing sub-basins is roughly the same. This is because the majority of the total population within transboundary river basins is located within few river basins.

In a nutshell, identifying and characterizing these river basins’ country-basin units in terms of their virtual water footprint could provide information that can be used for designing sustainable management schemes that take in to account not only the local water footprint and availability but also the global water footprint and availability.

## Figures and Tables

**Table 1:** The number of country-basin units which are blue, green, and gray virtual water importers and exporters and the number of people (millions) inhabiting these sub-basins. Period: 1996–2005.

Virtual Water Balance per Country−Basin Unit						
Blue Virtual Water Balance per Country−Basin Unit						
Range in km^3^	−26 to 21	−21 to −10	−10 to 0	0 to 2	2 to 6	
Number of Country−Basin Units	2	4	97	459	3	
Green Virtual Water Balance per Country−Basin Unit						
Range in Km^3^	−300 to 200	−200 to −40	−40 to 0	0 to 5	5 to 50	>50
Number of Country−Basin Units	1	7	187	339	30	1
Gray Virtual Water Balance per Country−Basin Unit						
Range in Km^3^	−50 to −10	−10 to −2	−2 to 0	0 to 4	4 to10	
Number of Country−Basin Units	4	13	156	390	2	
Total Virtual Water Balance per Country−Basin Unit						
Range in Km^3^	−400 to −30	−30 to −0	0 to −4	4 to −30	30 to −100	
Number of Country−Basin Units	10	193	322	37	3	
The number of country-basin units which imports and exports net virtual water and country−basin unit as well as area coverage and population count						
Virtual water	Export			Import		
	Number of Country−Basin Units	Population Number in Million	Area in million km^2^	Number of Country−Basin Units	Population Number in Million	Area in million km^2^
Blue	104	510.59	2718.25	461	1998.57	3681
Green	196	627.12	3358.5	369	1882.03	3041.25
Gray	174	649.08	3735	391	1860.08	3735
Total	204	739.67	3595.25	361	1767	2804.5

## Supplementary Materials

The following are available online at https://www.mdpi.com/1660-4601/16/5/703/s1, Figure S1. The total water footprint of production within transboundary river basins’ country-basin units. Period: 1996–2005. The water footprint of production for blue, green, and gray waters was calculated by aggregating the global water footprint obtained from Mekonnen and Hoekstra, 2011 [11] at 30 arc-minute resolution to a country-basin spatial resolution. This map was generated with ArcGIS 10.2 for desktop from Environmental Systems Research Institute (ESRI) Redlands, CA, USA [28], Figure S2. Blue water footprint of production within transboundary river basins’ country-basin units. Period: 1996–2005. The water footprint of production for blue, green, and gray waters was calculated by aggregating the global water footprint obtained from Mekonnen and Hoekstra, 2011 [11] at 30 arc-minute resolution to a country-basin spatial resolution. This map was generated with ArcGIS 10.2 for desktop from Environmental Systems Research Institute (ESRI) Redlands, CA, USA [28], Figure S3. The green water footprint of production within transboundary river basins’ country-basin units. Period: 1996–2005. The water footprint of production for blue, green, and gray waters was calculated by aggregating the global water footprint obtained from Mekonnen and Hoekstra, 2011 [11] at 30 arc-minute resolution to a country-basin spatial resolution. This map was generated with ArcGIS 10.2 for desktop from Environmental Systems Research Institute (ESRI) Redlands, CA, USA [28], Figure S4. Gray water footprint of production within transboundary river basins’ country-basin units. Period: 1996–2005. The water footprint of production for blue, green, and gray waters was calculated by aggregating the global water footprint obtained from Mekonnen and Hoekstra, 2011 [11] at 30 arc-minute resolution to a country-basin spatial resolution. (This map was generated with ArcGIS 10.2 for desktop from Environmental Systems Research Institute (ESRI) Redlands, CA, USA [28], Figure S5. The total water footprint of consumption within transboundary river basins’ country-basin units. Period: 1996–2005. This per capita water consumption per country-basin unit was determined by disaggregating the national consumption using the population count of the sub-basins. Then it was aggregated to the country-basin unit to determine the values at this spatial resolution. This map was generated with ArcGIS 10.2 for desktop from Environmental Systems Research Institute (ESRI) Redlands, CA, USA [28], Figure S6. Blue water footprint of consumption within transboundary river basins’ country-basin units. Period: 1996–2005. This per capita water consumption per country-basin unit was determined by disaggregating the national consumption using the population count of the sub-basins. Then it was aggregated to the country-basin unit to determine the values at this spatial resolution. This map was generated with ArcGIS 10.2 for desktop from Environmental Systems Research Institute (ESRI) Redlands, CA, USA [28], Figure S7. The green water footprint of consumption within transboundary river basins’ country-basin units. Period: 1996–2005. This per capita water consumption per country-basin unit was determined by disaggregating the national consumption using the population count of the sub-basins. Then it was aggregated to the country-basin unit to determine the values at this spatial resolution. This map was generated with ArcGIS 10.2 for desktop from Environmental Systems Research Institute (ESRI) Redlands, CA, USA [28]. Table S1: Blue, Green and Gray Virtual Water of Production within Global Transboundary River Basins per Country-Basin Unit in Km³/year. Period: 1996–2005; Table S2: Blue, Green and Gray Virtual Water of Consumption within Global Transboundary River Basins per Country-Basin Unit in Km³/year. Period: 1996–2005; Table S3: Blue, Green and Gray Virtual Water Balance within Global Transboundary River Basins per Country-Basin Unit in Km³/year. Period: 1996–2005.

## Appendix A. Net Virtual Water Footprint per Country-Basin Units.

The net virtual water footprint is the difference between the water footprint of consumption and production of an area. The net virtual water balance for transboundary river basins’ country-basin units is presented below and shown in Figure 2. In the figure, the variations among the country-basin units of the river basins are depicted.

In Africa, except for country-basin units of the Orange River in South Africa, the Limpopo in Mozambique and Zimbabwe, the Sabi in Mozambique and Zimbabwe, the Pungwe in Mozambique, the Zambezi in Tanzania, Angola, and Mozambique, the Ruvuma in Tanzania and Mozambique, the Congo in Tanzania, the Juba-Shibeli in Somalia, the Nile in Uganda, the Niger in Burkina Faso and Benin, the Guinea in Cameroon, Ivory Coast, and Nigeria, the Sanaga in Cameron, the Cross in Nigeria, the Mono in Togo, the Oueme in Benin, the Volta in Ghana and Togo, the Komoe and Sassandra in Ivory Coast, and the Gambia in Senegal, all the other country-basin units of transboundary river basins are net virtual water importers. The country-basin units of the continent with the highest net virtual water import are the sub-basin of the Nile in Ethiopia, Sudan, South Sudan, Kenya, and Egypt, the Lake Turkana, Awashm and Juba-Shibeli sub-basins in Ethiopia, the Zambezi in Zimbabwe, the Niger in Mali, the Volta in Burkina Faso, and the Limpopo in South Africa. In Asia, the country-basin units with largest net virtual water values are the Ganges-Brahmaputra-Meghna country-basin units, except for its sub-basin in China, the Bei Jiang/His and the Red/Song Hong Rivers’ sub-basins in China, the Indus in Pakistan, the Aral Sea in Tajikistan and Kyrgyzstan, and the Jenisej/Yenisey in Mongolia. The rest of the continents’ country-basin units are moderate importers and exporters of virtual water. In Eastern Europe, most of the country-basin units are net virtual water exporters while most of Western Europe’s country-basin units are virtual water importers, excluding the Douro/Duero, the Ebro and Guadiana in Spain, the Garonne in France, and the Guadiana in Portugal. This result is consistent with the results obtained by Vanham & Bidoglio, 2014 and Vanham, 2013 [16,17]. The same goes for the country-basin units of transboundary rivers in Northern Europe, except for the sub-basin of the Klaralven River in Sweden, which is a virtual water exporter. In North America, the Nelson-Saskatchewan and the Mississippi in the United States and Canada, and the Fraser and the Colombia in the United States are the main transboundary river sub-basins with negative net virtual water values.

In South America, the country-basin units of the Amazon in Peru, Colombia, Bolivia, and Ecuador, the Essequibo and Orinoco in Venezuela, the Corantijn/Courantyne and Maroni in Suriname, and the Lake Titicaca-Poopo System and La Plata in Bolivia are the net virtual water importing country-basin units in the continent.

This net virtual water footprint can be categorized as or disaggregated into blue, green, and gray net virtual water footprint. Even though, the aggregated virtual water balance map shows the variations among the transboundary river basins’ country-basin units it fails to depict the variations in terms of net blue, green, and gray virtual water balance among them. Disaggregating the net virtual water footprint can provide information about the blue, green, and gray consumption and production patterns, which might be indicative of, or consistent with, the socio-economic and environmental setting of the country-basin units. The following paragraphs identify and discuss the country-basin units of the main transboundary rivers, which are net blue, green, and gray water footprints importers and exporters.

River discharge is one of the sources of water supply for agricultural, industrial, and domestic water sectors. Transboundary rivers, considering the vast amount of area they cover, the huge number of people they include and due to the fact that they are responsible for more than half of the global runoff, they are among the main river basins which serve as sources of water for satisfying the water demands of these sectors. As the result identifying the surface water or blue virtual water balance of these border crossing rivers is vital especially at the country-basin spatial resolution.

In Africa, the Nile river basin’s sub-basins in Sudan and South Sudan are the sub-basins which are net blue virtual water exporters. Egypt largely depends on the river for supporting its agricultural, industrial, and domestic sectors. Hence the river’s sub-basin in Egypt is highly net blue virtual water importer. The other country-basin units in Ethiopia and Equatorial Africa are also net blue virtual water importers but not to the extent of the sub-basin in Egypt. Most of the continent’s transboundary rivers’ country-basin units are net blue virtual water importers, except the Niger sub-basin in Niger and Ivory Coast, the Limpopo and Orange in South Africa, the Juba-Shibeli in Somalia, the Awash in Ethiopia, the Zambezi in Malawi, the Okavango in Angola, the Incomati and Sabi in Zimbabwe, Lake Natron in Tanzania, the Daoura in Morocco, and the Komoe in Burkina Faso. In Asia country-basin units of the Ganges-Brahmaputra-Meghna, the Mekong River’s sub-basins in China, Cambodia, and Thailand, the Aral Sea in Kyrgyzstan, the Ili/Kunes He and Bei Jiang/Hsi in China, the Obxx and Oral/Ural in Kazakhstan, and th eHar Us Nur and Jenisej/Yenisey in Mongolia are all blue virtual water importers. The rest of the border crossing river basins’ country-basin units of the continents’ transboundary river basins are net blue virtual water exporters. In the Middle East country-basin units of the Kura-Araks in Turkey and Iran, the Coruh in Turkey, the Jordan in Jordan, the Asi/Orontes in Syria, and the Asi/Orontes in Turkey are the country-basin units which are net virtual blue water importers. In Europe, except the sub-basins of he Douro/Duero, Ebro, and Guadiana in Spain, the Garonne in France, the Tagus/Tejo and Guadiana in Portugal in Western Europe and the Dnieper and Don in Ukraine, the Don, Volga, Oral/Ural, and Obxx in Russia, in Eastern Europe all the other country-basin units of transboundary river basins are net virtual blue water importers.

In North America sub-basin of the Columbia, Mississippi, Colorado, and Nelson-Saskatchewan, the Rio Grande in the United States, the Fraser in Canada, and the Yaqui in Mexico are net exporters of the blue virtual water footprint. On the other hand, the sub-basins of the Rio Grande in Mexico, and the St. Lawrence and Yukon in the United States and Canada are net blue virtual water importers.

The net blue virtual water footprint of the Amazon in Brazil, Bolivia, Ecuador, and Colombia, Corantijn/Courantyne in Guyana, the Maroni in Suriname, and La Plata in Brazil and Paraguay are positive. As the result, they are net blue water footprint importers. La Plata in Bolivia, Uruguay, and Argentina, the Amazon in Peru, the Orinoco in Venezuela, and the Essequibo in Guyana are the country-basin units of transboundary river basins which are net blue virtual water exporters in the continent.

A large portion of the world’s population depends on rainwater or precipitation as a water supply for an agricultural sector, especially in developing countries. In Africa, the net green virtual water footprints of the Nile river basin’s country-basin units showed fewer variations among them. Most of the river’s country-basin units are net virtual water importers of rainwater. The sub-basin of the river in Uganda which is located in the equatorial region is the only net green virtual water exporter within the basin. Among the net green virtual water exporting country-basin units of transboundary river basins in the continent the main ones are the Juba-Shibeli in Somalia and Kenya, the Congo in Congo/Zaire, the Zambezi in Angola, the Ruvuma, Zambezi, and Limpopo in Mozambique, the Ruvuma in Tanzania, the Sabi and Limpopo in Zimbabwe, the Orange in South Africa, the Ogooue in Gabon, the Sanaga and Niger In Cameroon, the Niger in Nigeria, Guinea Burkina Faso, Ivory Coast, and Benin, the Volta in Ghana and Ivory Coast, and the Komoe in Ivory Coast. The other transboundary river basins’ country-basin units of the continent which are large net green virtual water importers other than the country-basin units of the Nile River are Limpopo in South Africa and the Niger in Mali.

In Asia country-basin units with the largest net green virtual water consumption are country-basin units of Ganges-Brahmaputra-Meghna except for the country-basin unit in China, the Indus in India and Pakistan, the Bei Jiang/Hsi in China, and the Aral Sea in Tajikistan and Uzbekistan. Tarim in China, sub-basin of the Mekong except the river’s sub-basin in Thailand, the Ganges-Brahmaputra-Meghna in China, the Jenisej/Yenisey in Mongolia, the Aral Sea in Kazakhstan, the Shu/Chu in Kazakhstan, the Ili/Kunes He and Pu Lun T’o in China, the Har Us Nur in Mongolia, and the Amur and Jenisej/Yenisey in Mongolia are also net green virtual water importing country-basin units but the magnitude of their net import is lower compared to the above country-basin units of transboundary rivers in the continent. In the Middle East the Kura-Araks in Armenia and Georgia, except for the country-basin units in Turkey and Iran, the Samur and Sulak in Russia, the Coruh in Turkey and the sub-basins of the Tigris-Euphrates/Shatt al Arab, except the one in Syria, are net green virtual water exporting basins.

In Eastern Europe, most of the country-basin units of transboundary river basins are net green virtual water exporters. The Danube in Austria, Croatia, Bosnia and Herzegovina, and Romania, the Maritsa in Bulgaria and the Vardar in Macedonia are the exceptions to this generalization. In Western Europe, most of the country-basin units of transboundary river basins are net green virtual water importers. The Ebro and Guadiana in Spain, the Garonne in France, the Douro/Duero, and the Tagus/Tejo Portugal are the country-basin units which are not net green virtual water importers in this part of Europe. In North America, the country-basin units of the Mississippi, Nelson-Saskatchewan, and Columbia in the United States and country-basin units of the Fraser basin are net green virtual water exporters. Sub-basins of the Yukon, Colorado, St. Lawrence, Rio Grande, and Yaqui are net green virtual water importing country-basin units in the continent.

In South America country-basin units of the Amazon in Peru, Ecuador, Colombia, and Bolivia, Orinoco in Venezuela and Colombia, La Plata in Bolivia, the Cancoso/Lauca in Chile and Bolivia, Lagoon Mirim in Brazil, the Maroni and Corantijn/Courantyne in Suriname, and the Essequibo in Venezuela are the sub-basin of transboundary river basins which have negative net green virtual water values.

When goods are traded among the different geographical areas they also carry gray water footprint of production associated with them. This is due to the fact that a specific volume of water which is specific to the kind of goods and production region is used to dilute the pollution associated with their production process. When these goods are traded the consuming area is responsible for the gray water footprint. Most of the country-basin units of Africa are net gray virtual water importers. The few exceptions are the Juba-Shibeli in Kenya and Somalia, the Zambezi in Angola and Mozambique, the Orange in Botswana and South Africa, the Sabi and Okavango in Zimbabwe, Senegal in Mali, the Niger in Guinea, Ivory Coast, and Burkina Faso, the Komoe in Burkina Faso and Atui Western Sahara and Mauritania. In Asia, the sub-basin of Amur in China, Indus in India and Pakistan have highly negative net gray water footprint of consumption. The country-basin units of the continent which are net gray virtual water importers are the Bei Jiang/His, Ganges-Brahmaputra-Meghna, and Mekong in China, the Aral Sea in Kyrgyzstan, Turkmenistan and Tajikistan, the Helmand, Hari/Harirud, and Kowl E Namaksar in Iran, and the Jenisej/Yenise, Har Us Nur, and the Amur in Mongolia. In the Middle East except for the Coruh in Turkey, the Jordan in Jordan, and he Asi/Orontes Syria the other transboundary river basins’ country-basin units are net gray virtual water exporters. In Eastern Europe, most of the country-basin sub-basins of transboundary river basins are net gray virtual water exporters. In Western Europe, except for the Douro/Duero and Guadiana in Spain, and the Garonne in France, the country-basin units of the other transboundary river basins are net gray virtual water importers. In Northern Europe the Vuoksa and Oulu in Finland, the Torne/Tornealven in Sweden, and the Glama in Norway are the net gray virtual water importing country-basin units.

In North America country-basin units of transboundary river basins with negative net gray virtual water values are the Mississippi in the United States and Canada, the Colombia in the United States and Canada, the Yaqui in Mexico, and the Fraser, St. Lawrencem and Yukon in Canada. On the contrary, the country-basin units of the Yukon, Yaqui, and St. Lawrence in the United States, and the Columbia in Canada are among the sub-basins with large net gray virtual water footprint. In South America the country-basin units with positive net gray virtual water footprint are Amazon in Colombia, Ecuador, and Bolivia, the Orinoco in Venezuela and Colombia, the Essequibo in Guyana and Venezuela, the Maroni in Suriname, the Cancoso/Lauca in Bolivia and Chile, and the Lake Titicaca-Poopo System in Peru and Bolivia are the main sub-basins of transboundary river basins that lie within the boundaries of the riparian countries that have positive net gray virtual water footprints. The rest of the river sub-basins are net gray virtual water exporters.
