# Supplementary material for: Assessment of Water Footprints of Consumption and Production in Transboundary River Basins at Country-Basin Mesh-Based Spatial Resolution
Source: Int J Environ Res Public Health. 2019 Feb 27;16(5):703. doi: 10.3390/ijerph16050703 (PMC6427118; doi:10.3390/ijerph16050703)
Supplement: Supplementary file 1 [file ijerph-16-00703-s001.zip › Supplementary material.doc]

# **Supplementary Results**

**Assessment of Water Footprints of Consumption and Production in Transboundary River Basins at Country-Basin Mesh-Based Spatial Resolution**

**Virtual Water Footprint of Production per Country-Basin Units**

From **Fig. S1** the variation in terms total water footprint of production among the country-basin units of the global transboundary river basins can be seen. In Africa, the water footprint of production for the sub-basins of the Nile in Sudan & South Sudan, sub-basins of the Niger in Nigeria and Niger are larger than the water footprints of the rivers’ other country-basin units and the rest of the continent’s transboundary river basins country-basin units. In Asia, the country-basin unit of Ganges-Brahmaputra-Meghna in India has a very big water footprint of production than the river’s remaining country-basin units and other country-basin units of the continent’s transboundary rivers. Other country-basin units with large water footprint of production than the rest of their country-basin units are Indus river basin in Pakistan and India, Bei Jiang/His River’s sub-basin in China and the sub-basin of Aral Sea in Uzbekistan. In Eastern Europe Donx, Volga and Obxx country-basin units in Russia have huge water footprint of production compared to the rest of country-basin unit of the continent. The Danube in Romania, the Dnieper in Ukraine, Vistula/Wista in Poland have also relatively bigger water footprint of production than their sub-basins within the other basin countries. In the Western and Northern Europe, the total water footprints of production within the country-basin units of transboundary river basins are relatively low and uniform.

**Figure S1.** The total water footprint of production within transboundary river basins’ country-basin units. Period: 1996–2005. The water footprint of production for blue, green and gray waters was calculated by aggregating the global water footprint obtained from (Mekonnen and Hoekstra, 2011) at 30 arc-minute resolution to a country-basin spatial resolution. This map was generated with ArcGIS 10.2 for desktop from Environmental Systems Research Institute (ESRI) ,Redlands, CA, USA,[28].

In North America, the sub-basins of the Mississippi in the United States have the largest water footprint of production. The Colombia and St. Lawrence country-basin units in the United States also have large water footprints of production. The Nelson-Saskatchewan country-basin unit in Canada has a larger water footprint of production than its sub-basin in the United States. In South America La Plata river basin’s sub-basins in Brazil and Argentina are the sub-basins in the continent with big water footprint of production compared to their remaining sub-basins in the other basin countries.

After disaggregating the total water footprint of production, variation in terms of the blue, green and gray water footprint of production among country-basin units can be observed. These variations arise because the sub-basins are different in terms of their socio-economic and environmental features. These variations are not depicted in the total water footprint of production map of country-basin units. **Fig. S2-S4** shows the disaggregated water footprints of production into blue, green and gray water footprints of production per country-basin units. In the paragraphs below the results are presented and the variations in terms of blue, green and gray water footprints of production among the country-basin units of the transboundary river basins are described.

In Africa the blue water footprints of production in the country-basin units of the Nile river basin in Sudan, South-Sudan and Egypt are larger compared with the rest of the river basin’s country-basin units. This is mostly due to large agricultural production within these country-basin units of the river. The blue water footprint of the production of the river’s sub-basins in Ethiopia and equatorial region is lower than the blue water footprints of the sub-basins in Sudan and Egypt. This might be due to the non-consumptive nature of water requiring economic activities in Ethiopia, mainly hydroelectric power generation and the abundance of internal water resources in the country-basin units of the river in equatorial riparian countries. The country-basin units of the Orange and Limpopo river basins in South Africa also show large blue water footprint of production compared with the rest of the country-basin units of the other border crossing rivers of the continent. The other transboundary river basin’s country-basin units of the continent do not show a big difference in terms of the blue water footprint of production. This water footprint of production is annual but if the water footprint accounting is done seasonally we might observe variations among the country-basin units. This is mainly because agricultural production which is the major user of water varies seasonally within the river basins. In Asia, the Ganges-Brahmaputra-Meghna in India and Indus in India & Pakistan have very huge annual blue water footprints of production. This could be mainly due to all year round water-intensive agricultural practice. While the river’s sub-basins in China, Nepal, and Bangladesh have relatively small annual water footprint of production. Tarim Rivers’ country-basin unit in China and the Aral Sea’s country-basin unit in Uzbekistan also shows large annual water footprints compared to their country-basin units in the other sharing countries. Furthermore, the sub-basin of the Mekong in Thailand also have larger annual blue water footprint of production than the rest of the country-basin units. In the Middle East, Tigris-Euphrates/Shatt al Arab sub-basin in Iraq have large blue water footprint of production compared to the river’s other sub-basins within the rest of the riparian countries. Kura-Araks river’s sub-basins in Iran and Azerbaijan also have bigger blue virtual water footprints of production than it’s sub-basins within Georgia and Armenia borders. In Europe, the Volga River’s country-basin unit in Russia supports very large annual blue water footprint of production. The Donx River’s sub-basin in Russia, Dnieper sub-basin in Ukraine, Danube’s sub-basin in Romania, Rhine & Elbe in Germany, Po in Italy, Seine in France, Douro/Duero, Guadiana & Ebro in Spain also showed large annual blue water footprint of production relative to their country-basin units in the other sharing states. Within the transboundary river basins’ country-basin units of Northern Europe, the blue virtual water footprint of production is more or less uniform.

In North America, the country-basin units of the Mississippi, Colombia & Colorado River basins in the United States have large annual blue water footprint of production. The water footprint of St. Lawrence country-basin units in the United States and Canada as well as Rio Grande in Mexico & United States and Nelson-Saskatchewan in the United States also have very large annual blue water footprint of production than their remaining country-basin units also but less than the water footprints of Mississippi, Colombia, Colorado, Yaqui and Rio-Grande country-basin units in the United States. In South America, the country-basin units of the La Plata river basin in Argentina and Brazil, Amazon River’s country-basin unit in Peru and Orinoco in Venezuela have annual blue water footprint of production is bigger than the remaining rivers’ sub-basins in the rest of the riparian countries.

**Figure S2.** Bluewater footprint of production within transboundary river basins’ country-basin units. Period: 1996–2005. The water footprint of production for blue, green and gray waters was calculated by aggregating the global water footprint obtained from (Mekonnen and Hoekstra, 2011) at 30 arc-minute resolution to a country-basin spatial resolution. This map was generated with ArcGIS 10.2 for desktop from Environmental Systems Research Institute (ESRI) ,Redlands, CA, USA,[28].

The following section will highlight the variation among country-basin units of transboundary river basins in terms of their rainwater or precipitation use for production of goods and services. This can assist for allocation of the rivers contested discharge taking in to account the precipitation usage differences for production purposes among global transboundary river basins’ country-basin units.

In Africa the green water footprint of production for the country-basin units of the Nile river basin in the equatorial region, Ethiopia and Sudan are relatively the same. While in the sub-basin of the river in Egypt the virtual blue water footprint of production is lower than the rest of the river basin’s country-basin units. This might be because the sub-basin in Egypt is located in the arid geographical zone. In Western Africa, the country-basins of the Volta in Ghana, Niger & Lake Chad’s sub-basins in Nigeria have green water footprints of production which are greater than the rest of the basin’s sub-basins in the other riparian countries. The sub-basin of the Congo River in Congo and the Orange River basin’s sub-basin in South Africa the green water footprints of production are much higher. In Asia, the Ganges-Brahmaputra-Meghna in India consumes more green water for production than its sub-basin in China, Bhutan, Bangladesh, and Nepal. The sub-basin of the Indus in India also have higher green water footprint of production than its sub-basin in China and Pakistan. In the Mekong, the river’s sub-basin in Thailand consumes more rainwater for production than the sub-basins of the basin in China, Laos, and Cambodia. Green water footprint for production in Bei Jiang/His River’s sub-basin in China is greater than its sub-basin in Vietnam. Amur in China and Russia, as well as the Obxx river basin in Russia and Kazakhstan, are also among the sub-basins of transboundary river basins in Asia that have larger green water footprint of production than the rest of their sub-basins.

In Europe, except for Dnieper River’s sub-basin in Ukraine and Donx river basin in Russia, the other river basins show very little disparity in terms of green water footprints of production. The Dnieper in Ukraine, Donx, Obxx, and Volga in Russia are the sub-basins which have huge rainwater dependency for production. The Danube in Romania and Hungary, Oder/Odra and& Vistula/Wista in Poland, the Elbe in Germany and, Dnieper in Belarus and& Russia, and the Seine in France also have slightly bigger green water footprint of production than the rest of their sub-basins in the other riparian states. In North America, Mississippi and& Columbia in the United States, and Nelson-Saskatchewan in Canada have larger rainwater consumption than the rest of their country-basin units. In South America the green water footprint of production for Amazon River’s basin in Brazil and La Plata river basin in Argentina, Paraguay and Brazil have relatively bigger green water footprint of production than their and other river basins’ country-basin units in the continent. In the La Plata river basin, the order of the river’s country-basin units in terms decreasing order of green water footprint of production is the river’s sub-basin in Brazil followed by the sub-basins in Argentina and Paraguay respectively.

**Figure S3.** The green water footprint of production within transboundary river basins’ country-basin units. Period: 1996–2005. The water footprint of production for blue, green and gray waters was calculated by aggregating the global water footprint obtained from (Mekonnen and Hoekstra, 2011) at 30 arc-minute resolution to a country-basin spatial resolution. This map was generated with ArcGIS 10.2 for desktop from Environmental Systems Research Institute (ESRI) ,Redlands, CA, USA,[28].

Gray water footprint of production is the amount of water used to dilute the pollution which resulted in the process of producing goods and services. Within the country-basin sub-basins of Africa’s transboundary river basins, the production footprint of gray water is not high. Furthermore, the variation among the transboundary rivers’ sub-basins is not that big. The sub-basin of the Nile river basin in Egypt is an exception to this generalization because it has large gray water footprint of production than the rest of the rivers’ sub-basins. In Asia the sub-basins of the Indus in India and Pakistan, the sub-basin of Ganges-Brahmaputra-Meghna in India, Amur & Bei Jiang/His River’s sub-basins in China and Mekong in Cambodia have bigger water footprint of production than their other sub-basins in their other riparian countries and country-basin units of the rest of the transboundary river basins in the continent. Rhine, Elbe and& Rhine in Germany, the Seine in France, Po in Italy in are the country-basin units in Western Europe that have larger water footprint of production than their other country-basin units. On the other hand in Easter Europe, Vistula/Wista and Oder/Odra in Poland, the Dnieper in Ukraine Danube in Romania & Hungary, and the Volga, Jenisej/Yenisey, & Obxx in Russia have larger gray water footprint of production than the rest of their sub-basins. In North America, the sub-basins of the Mississippi have huge gray water footprint of production. The Colombia & St. Lawrence in the United States and Nelson-Saskatchewan in Canada also have relatively bigger gray water footprint of production in the continent.

In South America, the country-basin unit of the La Plata river basin in Brazil is larger the than the river’s other country-basin units in the other basin countries. The other country-basin units of the continent have uniform water footprint of production throughout their country-basin units.

**Figure S4.** Gray water footprint of production within transboundary river basins’ country-basin units. Period: 1996–2005. The water footprint of production for blue, green and gray waters was calculated by aggregating the global water footprint obtained from (Mekonnen and Hoekstra, 2011) at 30 arc-minute resolution to a country-basin spatial resolution. This map was generated with ArcGIS 10.2 for desktop from Environmental Systems Research Institute (ESRI) ,Redlands, CA, USA,[28].

**Virtual Water Footprint of Consumption per Country-Basin Units**

The country-basin units of the Niger in Niger and Nigeria, the sub-basin of the Nile in the Egypt, Sudan, South-Sudan, and Ethiopia are the country-basin units of the transboundary river basins with the largest water footprint of consumption in the continent. In the rest of the country-basin units of the continent, the water footprints of consumption are not that big. In Asia, the country-basin units of the Ganges-Brahmaputra-Meghna in India, Indus river basin in Pakistan and India, Amur and Bei Jiang/His in China have large total water footprints of consumption. Country-basin unit of the Aral Sea in Uzbekistan, country-basin unit of the Ganges-Brahmaputra-Meghna in Nepal and Bangladesh, Irrawaddy in Myanmar (Burma) and the Mekong in Thailand follows the above country-basin units in terms of the magnitude of the water footprint of consumption. In the Middle East, the water footprints of consumption are uniform throughout the country-basin units of the region’s transboundary river basins. In Eastern Europe, the Dnieper in Ukraine, the Danube in Romania, Vistula/Wista Volga and Obxx in Russia have larger water footprints of consumption than the rest of their sub-basins. In Western Europe sub-basins of Poxx in Italy, the Rhine in Germany and Seine in France have the larger total water footprint of consumption than the rest of their sub-basins in the remaining riparian countries. In North America, the total water consumption for Mississippi in the United States and St. Lawrence in the United States and Canada have a large total footprint of consumption. In South America, the sub-basin the La Plata in Brazil have greater water footprints of consumption than its sub-basins in the other sharing countries. The other transboundary river basins’ country-basin units in the continent have uniform water footprint of consumption throughout the basins.

**Figure S5.** The total water footprint of consumption within transboundary river basins’ country-basin units. Period: 1996–2005. This per capita water consumption per country-basin unit was determined by disaggregating the national consumption using the population count of the sub-basins. Then it was aggregated to the country-basin unit to determine the values at this spatial resolution. This map was generated with ArcGIS 10.2 for desktop from Environmental Systems Research Institute (ESRI) ,Redlands, CA, USA,[28].

In order to understand the water footprints of consumption in more detail desegregation of the total water consumption footprint into blue, green and gray water footprint of consumption is important. **Fig. S6-S8** in the following section shows the disaggregated water footprint of consumption into blue, green and gray water footprints of consumption.

In the following paragraphs variations in terms of the water footprint of consumption among the country-basin units of transboundary river basins are presented. The blue water footprint of consumption refers to the domestic and international surface water or river discharge resources directly and indirectly consumed by the inhabitants of an area.

In Africa the sub-basin of the Nile River in Sudan, South-Sudan and Egypt the blue water footprint of consumption is large. With the sub-basin of the river in Egypt having bigger blue water footprint of consumption than the other country-basin units of the river basin and the rest of the country-basin units of the transboundary rivers of the continent. Niger River’s sub-basin in Mali, Niger, and Nigeria also have greater blue water footprint of consumption but less than the Nile river basin’s country-basin units. In Asia, the sub-basin of the of Ganges-Brahmaputra-Meghna in India, Indus in Pakistan and Bei Jiang/His in China have large blue water footprint of consumption while the Aral sea’s sub-basin in Uzbekistan, Amur river in China and Mekong river in Thailand follows them. In the Middle East, the Euphrates and Tigris river basin in Iran have greater blue water footprint of consumption followed by the river’s sub-basins in Turkey and Syria. While the blue water footprint of consumption within the part of the basin Iraq is not that large compared to the other sub-basins of the river.

**Figure S6.** Bluewater footprint of consumption within transboundary river basins’ country-basin units. Period: 1996–2005. This per capita water consumption per country-basin unit was determined by disaggregating the national consumption using the population count of the sub-basins. Then it was aggregated to the country-basin unit to determine the values at this spatial resolution. This map was generated with ArcGIS 10.2 for desktop from Environmental Systems Research Institute (ESRI) ,Redlands, CA, USA,[28]..

In Western Europe except for the sub-basins of Douro/Duero in Spain, Garonne in France, Tagus/Tejo in Portugal the other county-basin of the region’s transboundary rivers blue water footprint of consumption is large. In Eastern Europe, the sub-basins of Oder/Odra and Vistula/Wista in Poland, the Dnieper in Ukraine, the Danube in Romania, Obxx, Donx & Volga in Russia are the main country-basin units of transboundary river basins with large blue water footprint of consumption. In North America, the sub-basin of the Mississippi in the United States has a very large blue water footprint of consumption. The sub-basins of the Colombia & Colorado in the United States and the Rio Grande in the United States and Mexico also have large blue water footprint of consumption but relatively less than that of the Mississippi. In South America in the sub-basins of the Amazon in Peru & Venezuela, the Orinoco in Venezuela and the sub-basins’ of the La Plata river basin in the Argentina & Brazil the blue water footprints of consumption are larger than the blue water footprints of consumption within their other country-basin units.

In Africa country-basin units of Niger in Niger & Nigeria, sub-basin of the Nile in Sudan and South-Sudan are the country-basin units with large green water footprint of consumption in the continent. Orange & Limpopo in South Africa, Congo sub-basin in Congo, the sub-basin of the Nile in the equatorial region and Lake Chad in Chad have slightly larger green water footprint of consumption than their other country-basin units. In Asia, the sub-basin of the Ganges-Brahmaputra-Meghna in India have larger green water footprint of consumption than the rest of the river’s sub-basins. The green water footprint of consumption of the Indus river basin in Pakistan is also larger than the footprints of its sub-basins in India and China. The rainwater dependence of the country-basin unit of Amur River basin in China is larger than the dependence of river’s other sub-basin in that lies within the border of Russia. Other country-basin units with large green water footprint of consumption are the Mekong river basin’s sub-basins in Cambodia and Thailand. In the Middle East, the country-basin units of Euphrates and Tigris in Turkey, Syria, and Iran is larger than the rainwater consumption footprint of the sub-basin of the river in Syria. In Eastern Europe, the Volga & Obxx in Russia, the Danube in Romania are the border crossing river basins’ sub-basins with slightly larger water footprint of consumption than their remaining sub-basins. In Western Europe Rhine in Germany, Po in Italy and Tagus/Tejo in Spain are the transboundary river basins’ sub-basins with larger green water footprint of consumption than the rest of their sub-basins within their other riparian states.

**Figure S7.** The green water footprint of consumption within transboundary river basins’ country-basin units. Period: 1996–2005. This per capita water consumption per country-basin unit was determined by disaggregating the national consumption using the population count of the sub-basins. Then it was aggregated to the country-basin unit to determine the values at this spatial resolution. This map was generated with ArcGIS 10.2 for desktop from Environmental Systems Research Institute (ESRI) ,Redlands, CA, USA,[28].

In Northern America, the sub-basin of the Mississippi in the United States have far larger green water footprint of consumption than its sub-basin in Canada. The sub-basin of Rio Grande (North America) have lower green water footprint of consumption than the river’s sub-basin in Mexico. The Colombia, the Colorado and St. Lawrence sub-basins in the United States also have bigger rainwater footprint of consumption than their remaining sub-basins. The Orinoco in Venezuela, Amazon in Brazil & Bolivia and LaPlata in Brazil, Argentina & Paraguay are country-basin units of the main border crossing river basins in South America with big water footprint of consumption.

The water consumption footprint of river basins also partly Gray water footprint of consumption. This section will explain the differences in terms of gray water consumption for the main transboundary river basin within the continents. In Africa, the sub-basin of the Nile in Egypt, Niger in Nigeria, Limpopo, and Orange in South Africa are sub-basins of transboundary river basins with large gray water footprint of consumption. The rest of the transboundary river basins’ country-basin units have lower but more or less uniform gray water footprint of consumption throughout the continent. In Asia, very large gray water footprint of consumption were observed in the sub-basins of Ganges-Brahmaputra-Meghna in India, Bangladesh & China, Indus river basin in Pakistan & India, Amur & Bei Jiang/His in China, Mekong river basin in Thailand. Other country-basin units in the continent like the Tarim River in China, Aral Sea’s sub-basin Uzbekistan also have water footprint which is slightly greater than their other country-basin units. In the Middle East, the sub-basin of the Euphrates and Tigris river basin in Turkey, Syria, and Iran have similar gray water footprint consumption while the sub-basin in Iraq has the lowest gray water footprint of consumption in the basin.

In Eastern Europe Dniester in Moldova & Ukraine, Danube in Ukraine and Austria, Germany, Slovakia, Czech Republic & Hungary, Rhine in Switzerland, Rhone in France, Ebro & Tagus/Tejo in Spain have slightly lesser gray water footprint of consumption than their remaining country-basin units as well as the other country-basin units of the continent. On the other hand Dnieper in Ukraine, Vistula/Wista & Oder/Odra in Poland, the Rhine in Germany, the Seine in France and Volga & Donx in Russia are the other sub-basins of transboundary river basins with large gray water footprint of consumption. In North America, the Gray water footprint of consumption for the Mississippi River in the United States is far bigger than the other country-basin units of the continent’s transboundary rivers. The country-basin unit of the Colombia and Colorado, as well as St. Lawrence in the United States, also have large gray water footprint of consumption compared with their other country-basin units. In South America, the LaPlata river basin’s sub-basin in Brazil has the large gray water footprint of consumption than its sub-basins in Paraguay and Argentina. In the Amazon River basin the river’s sub-basins in Colombia, Bolivia, Ecuador, and Guyana have low gray water footprint of consumption than the rest of the rivers’ sub-basins located within the boundaries of the other riparian countries. While Lake Titicaca-Poopo System sub-basins, Essequibo in Guyana, Amazon sub-basins in Peru and Colombia have low gray water footprint of consumption compared with the country-basin units of the continents country-basin units stated above.

**Figure S8.** Gray water footprint of consumption within transboundary river basins’ country-basin units. Period: 1996–2005. This per capita water consumption per country-basin unit was determined by disaggregating the national consumption using the population count of the sub-basins. Then it was aggregated to the country-basin unit to determine the values at this spatial resolution. This map was generated with ArcGIS 10.2 for desktop from Environmental Systems Research Institute (ESRI, Redlands, CA, USA,[28].
